# Surgical approach to intraabdominal sepsis: a damage control approach can reduce stoma rates

**DOI:** 10.1007/s00464-025-12303-1

**Published:** 2025-10-20

**Authors:** Héloïse Smet, Jeanne Hirt, Dieter Hahnloser, Fabian Grass

**Affiliations:** Department of Visceral Surgery, Lausanne University Hospital CHUV, University of Lausanne (UNIL), Rue du Bugnon 46 – BH 10-222, 1011 Lausanne, Switzerland

**Keywords:** Staged, Damage control, Complicated diverticulitis, Two-stage

## Abstract

**Background:**

In patients with perforated diverticulitis and/or intraabdominal sepsis (IAS), the best surgical strategy remains controversial. While open, non-restorative Hartmann’s procedure represents the procedure of choice in many centers, ostomy-sparing and minimally invasive techniques have been increasingly utilized due to short- and long-term benefits, including a reduced stoma rate. This dynamic manuscript illustrates strategic and technical considerations of the standardized institutional staged or damage control approach to IAS.

**Technique:**

The institutional strategy to IAS is demonstrated through an emergency procedure for perforated Hinchey IV diverticulitis in a 70-year-old woman developing an acute abdomen. After a diagnostic laparoscopy, stercoral peritonitis was confirmed and the patient underwent laparotomy. A two-stage approach (stage 1: damage control with resection of the perforated sigmoid, abdominal lavage, vacuum-assisted closure (VAC) – stage 2 (36–48 h later and during daytime): primary colorectal anastomosis and abdominal closure) was performed. While laparoscopic exploration represents the standard approach in hemodynamically stable patients, the staged procedure (resection and sepsis control) helped refine the definite strategy (goal of reconstruction and no ostomy) in a clinically improved patient during the second step. The patient recovered well during the critical therapeutic window, allowing to perform primary anastomosis and a stoma-free discharge.

**Discussion:**

Clinical condition during surgery, surgeon expertise, and comorbidities are key determinants to decide whether to proceed to primary anastomosis in the case of IAS. An institutional series of our group revealed feasibility and safety of the staged approach, with 65% stoma-free patients at discharge. A standardized algorithm based on intraoperative hemodynamic parameters intends to guide surgical decision-making during staged procedures to seek optimized conditions allowing to proceed to primary anastomosis.

**Conclusion:**

A staged approach according to a predefined decisional framework may help decrease the need for ostomy creation in patients with IAS.

**Supplementary Information:**

The online version contains supplementary material available at 10.1007/s00464-025-12303-1.

Intraabdominal sepsis (IAS) accounts for over 10% of sepsis cases presenting to emergency departments and is associated with high mortality, with in-hospital rates ranging from 23 to 38% [1–3]. Rapid and effective source control remains a cornerstone to effectively manage these critically ill patients [2].

Concurrently, the incidence of diverticulitis is increasing, leading to a parallel rise of cases with IAS secondary to perforated diverticulitis [4, 5]. Historically, the standard treatment consisted of an open Hartmann’s procedure. However, with the development of the concept of the damage control surgery (DCS) in severe trauma management, DCS seems also an appropriate approach for patients with IAS [6, 7].

One of the advantages of this procedure is the reduction of the stoma rate formation [8]. End colostomies substantially decrease quality of life, with reversal procedures performed in only 50% of patients [9, 10]. In an attempt to reduce the rate of end ostomies and increase the rate of primary anastomosis, our group implemented the concept of a staged approach in the setting of severe IAS [11]. In the light of the institutional experience and recently published guidelines mentioning the staged approach as a potential alternative, the aim of this article was to illustrate and discuss the institutional algorithm of a staged procedure [12].

## Procedure

The staged approach is illustrated in a 70-year-old woman with a known history of arterial thrombosis secondary to primary myelofibrosis, for which she had previously undergone a peripheral arterial bypass. Her ongoing treatments included an antivitamin K anticoagulant and Ruxolitinib, a JAK1/2 inhibitor with immunomodulatory properties.

The patient presented to the emergency department of our tertiary care center with acute onset abdominal pain and nausea. Laboratory tests revealed leukocytosis with a normal C-reactive protein level. The international normalized ratio (INR) was elevated at 3.7. Lactate levels were within normal limits (1.6 mmol/L), and the remainder of the metabolic panel was unremarkable.

An abdominal CT scan revealed diffuse retroperitoneal air, and a moderate pneumoperitoneum associated with free peritoneal fluid due to perforated diverticulitis. Empiric broad-spectrum antibiotic therapy was initiated. On clinical examination, the patient exhibited signs of peritoneal irritation but remained afebrile and hemodynamically stable. A hematology consult was sought to clarify her immunologic situation to reduce surgical risk. The patient was hospitalized under very close surveillance in the intermediate care unit. Twenty-four hours after admission, the patient became febrile with increasing abdominal pain. Tachycardia was observed with normal tension values. Due to the obvious development of IAS, she was immediately taken to the operating room at 09:00 PM (Supplemental Video).

At laparoscopic exploration, stercoral peritonitis was found in the lower abdomen due to a large perforation of the sigmoid colon for which sigmoidectomy was indicated. Due to increasing hemodynamic instability and the presence of feces, conversion to a midline laparotomy was decided. A non-oncologic mesenteric dissection was carried out. The upper rectum and descending colon were transected using a stapling device. The abdomen was rinsed with 6 L of sterile saline. Hemodynamic parameters at this stage were as follows:

- Lactates: 2.94 (normal range: 0.5 – 2.2 mmol/l)
- Norepinephrine: 15 mcg/min
- pH: 7.38 (normal range: 7.35–7.45)
- base excess (BE): − 7.3 (normal range: − 2 – 2 mEq/l)

It was decided not to reconstruct at this moment and a vacuum-assisted closure (VAC) device was applied. The patient was transferred to the intensive care unit (ICU) for resuscitation with a planned 2nd-stage procedure 36–48 h later.

48 h later, the patient was hemodynamically stable allowing a second stage during daytime, organized in the presence of a board-certified colorectal surgeon. The operation revealed a clean abdomen. Given the favorable local condition and hemodynamic stability, an end-to-end anastomosis was performed using a standard 29 mm circular stapling device. Air-leak test was favorable, and one drain was placed in the pelvis before definitive abdominal closure. According to institutional protocol, a subcutaneous VAC dressing was placed due to the diagnosis of stercoral peritonitis at first stage [13].

Postoperative recovery was uneventful. The subcutaneous wound was closed in two steps. The first step was performed in the operating room, three days after the anastomosis, with partial wound closure. Complete closure was achieved at the bedside three days later. Antibiotic therapy was continued for 10 days total. Anticoagulation was rapidly reintroduced, and the patient was discharged on postoperative day (POD) 12. At three months of follow-up, the patient was doing fine with no complications.

## Technical considerations

### Diagnostic laparoscopy

In our institution, laparoscopic exploration represents the first-choice approach unless contraindicated (hemodynamics impeding tolerance of pneumoperitoneum, comorbidities, surgical history) upon discussion with anesthesiology in case of suspected IAS and surgical expertise. The procedure is carried out using standard patient positioning in lithotomy and trocar setup. Following an open view approach, all four abdominal quadrants are explored to assess the severity of contamination. Extensive peritoneal lavage (source control) is then carried out to control purulent and fecal contamination, using a total of at least 6 L of sterile saline. Gentle manipulation using a suction device is warranted due to the possible fragility of inflamed bowel tissue.

### Identification of source of sepsis

The next step consists of identifying the source of IAS. In case of Hinchey III peritonitis without fecal contamination and no visible perforation (we do not search for the perforation by rectal injection), extensive adhesiolysis should be avoided and a laparoscopic lavage procedure can be considered [14]. If during exploration a small perforation is found, it can be sutured and covered with adjacent fat tissue or omentum.

### Limited resection (damage control of the septic source)

Identification of an overt perforation and/or fecal peritonitis warrants surgical resection of the perforated segment. In cases of IAS, mobilization should be limited to the affected bowel segment to allow a segmental resection; full mobilization of the descending colon and splenic flexure should be avoided, as it may complicate the second operation. The objective is to achieve resection of the perforated segment and peritoneal lavage within 60 to 90 min, followed by patient stabilization in the ICU. Gastric decompression with a nasogastric tube and initiation of parenteral nutrition should be implemented at the end of the initial operation. In the presence of a stercoral peritonitis without IAS, we usually proceed in a two-step approach to increase the chance of stoma-free discharge.

### Stabilization of the patient

**Fig. 1:**
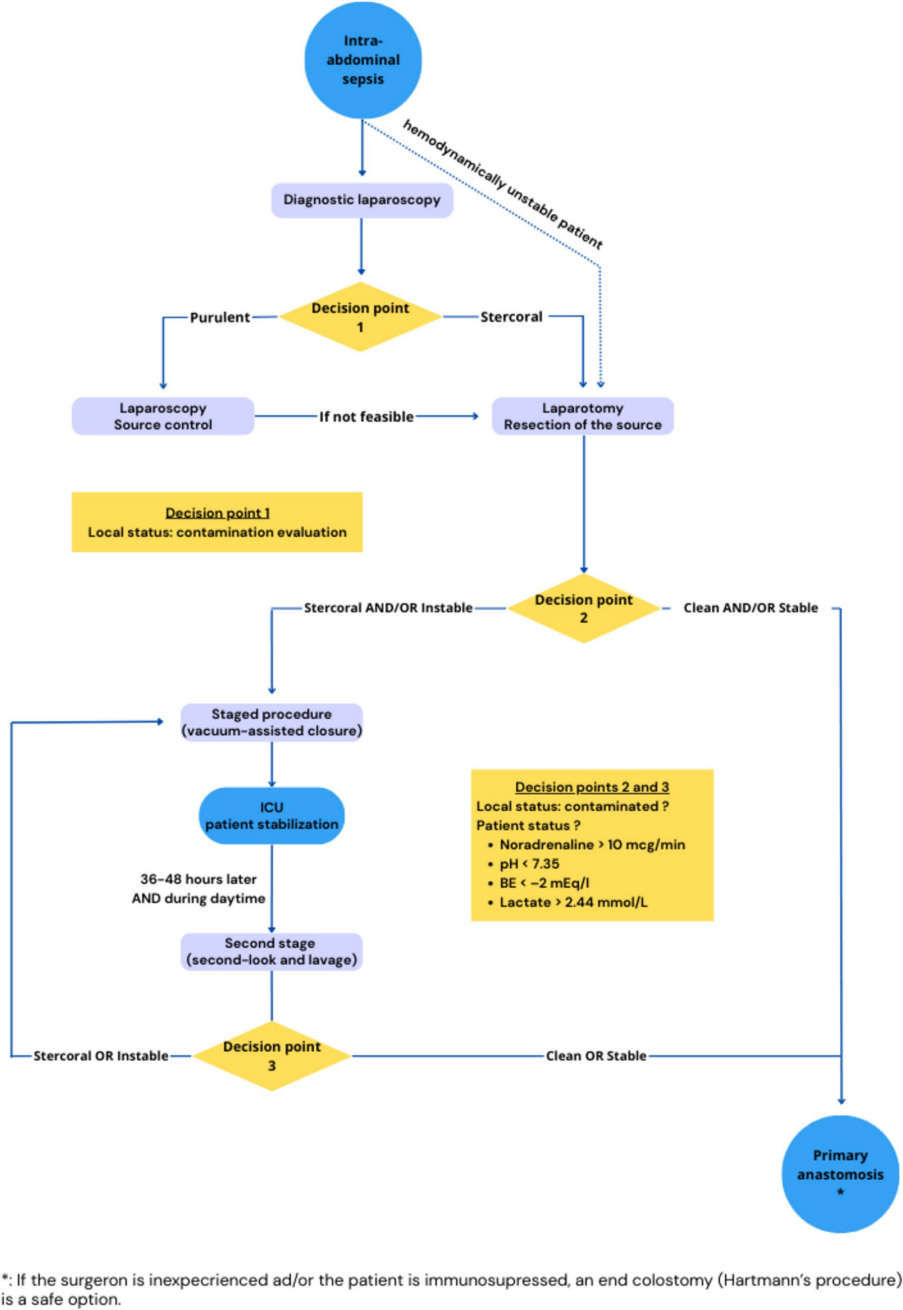
Treatment algorithm : standardized treatment approach for patient with intraabdominal sepsis in case of perforated diverticulitis. *BE* Base excess, *ICU* intensive care unit

At this stage, the decision to proceed to a staged approach, consisting of abandoning the stumps while applying an abdominal VAC, is taken according to institutional protocol and predefined hemodynamic criteria (noradrenaline, pH, lactates, and base excess (BE), Fig. 1). The VAC device is maintained for 36–48 h, allowing hemodynamic resuscitation during this interval period, aiming to improve the likelihood to proceed to delayed anastomosis under optimized conditions.

### Second-stage operation and reconstruction

The 2nd-stage operation is scheduled 36 to 48 h later and during daytime hours to ensure availability of all resources and presence of a board-certified (European Board of Surgery Qualification in Coloproctology) colorectal surgeon. Exploration is initiated with repeated lavage of the abdominal cavity. In case of persistent IAS and/or peritonitis, a 3rd stage can be considered 24 h later. Otherwise, colorectal anastomosis is performed through an end-to-end or side-to-end technique with a circular stapling device upon surgeon’s discretion. Anastomotic integrity is tested, and the abdominal cavity is closed, placing at least one abdominal drain in the pelvis. Close monitoring is required and, in case of deterioration, a low threshold for surgical re-exploration (re-lavage) rather than performing computed tomography (CT) imaging is advocated.

Broad-spectrum antibiotics are initiated shortly after diagnosis of IAS and continued for 10 days, in accordance with institutional protocol. We recommend the use of Piperacillin–Tazobactam as first-line agent. Intraoperative cultures are obtained at each surgical intervention to tailor antibiotic therapy. This is particularly important given the association of specific pathogens with poorer outcomes [15, 16]. Consultation with infectious disease specialists may be sought given the complexity and potential for multidrug-resistant organisms in these cases.

## Discussion

Currently, intraoperative treatment decisions when confronted with complicated diverticulitis are guided by the degree of contamination according to the Hinchey classification [17]. However, the optimal surgical approach remains a subject of debate. According to recent guidelines of the European Association for Endoscopic Surgery (EAES) and the European Society for Coloproctology (ESCP), primary anastomosis is recommended in cases of complicated diverticulitis without signs of sepsis, provided the procedure is performed by surgeons with expertise in colorectal surgery [12]. Conversely, in the presence of IAS, absence of an experienced colorectal surgeon, or in patients who are frail or immunocompromised, a Hartmann’s procedure remains the standard. In selected cases of purulent peritonitis, laparoscopic peritoneal lavage may be a valid option. Fecal peritonitis or identification of an overt sigmoid perforation mandate colonic resection. Instead of deciding whether primary anastomosis or Hartmann’s procedure should be performed, a staged approach as illustrated in the present manuscript may represent a 3rd option in this setting.

With the emergence of the DCS concept in trauma care, its application to IAS has significantly increased over the past decade. In 2010, Perathoner et al. were among the first to describe its use in the context of perforated diverticulitis [18]. Their group compared three surgical strategies: (I) resection with primary anastomosis, (II) limited resection with blind closure of colonic ends and temporary abdominal vacuum-assisted closure, followed by a second-stage procedure to either restore continuity or create an end colostomy, and (III) the classical Hartmann’s procedure. The choice of approach was based on intraoperative findings and surgeons’ discretion. Key decision-making factors included patient comorbidities, hemodynamic instability, and intraoperative condition. The authors emphasized that a second-stage procedure enabled anastomosis to be performed during daytime hours and under the care of a colorectal surgeon––an important consideration, as after-hours anastomoses have been associated with a higher risk of leakage [19].

More recently, a meta-analysis of randomized clinical trials (RCTs) evaluating DCS for complicated diverticulitis demonstrated restoration of gastrointestinal continuity in 62.1% of patients [6]. The rates of major anastomotic leak and overall mortality were 4.7% and 9.2%, respectively. These outcomes are consistent with previously reported data [20, 21] supporting the safety of DCS in this context, with the advantage of a stoma-free discharge.

Some authors have proposed algorithms incorporating hemodynamic instability to guide two-stage surgical management in selected patients [22]. However, accurately defining hemodynamic stability in the setting of emergency surgery remains challenging.

In a consecutive case series of staged procedures for IAS, our institution proposed 4 intraoperative criteria with respective thresholds: noradrenaline > 10 µg/min, pH < 7.35, base excess < –2 mEq/l, or lactate > 2.44 mmol/L [11]. In such cases, damage control surgery as a 1st step using a vacuum device for transient abdominal closure if available is advised. This staged approach allows to reassess the situation in the presence of experienced surgical and anesthetic staff. The principal aim of this strategy consists of decreasing the necessity of ostomy creation. In a previous institutional series, this strategy allowed 65% of patients to be stoma-free at discharge after IAS including complicated diverticulitis, while other studies consistently reported an ostomy rate of up to 75% in case of one-step approach in similar settings [23]. We have further shown that a third or fourth look is feasible and safe and may increase anastomosis rate [11].

Our group implemented an algorithm for management of IAS (Fig. 1). While the surgical approach (open or minimally invasive) is left to the surgeon’s discretion based on the patient’s clinical presentation, medical history and experience with either approach, the algorithm provides guidance for management of IAS.

In the present case, a two-stage approach was selected due to ongoing IAS in the presence of a stercoral peritonitis. The patient improved considerably during the 48-h resuscitation period. During the 2nd-stage procedure, an end-to-end anastomosis was safely performed in the presence of a board-certified colorectal surgeon.

## Conclusion

This dynamic manuscript intends to illustrate both the feasibility and safety of a staged approach to IAS in the setting of perforated diverticulitis. A staged approach using predefined criteria for intraoperative guidance may help to reduce the necessity for ostomy creation and increase stoma-free discharge of the patients.

## Supplementary Information

Below is the link to the electronic supplementary material.

Supplementary file1 (MP4 328841 KB)

## References

[1] Bova R, Griggio G, Vallicelli C, Santandrea G, Coccolini F, Ansaloni L, Sartelli M, Agnoletti V, Bravi F, Catena F (2024) Source control and antibiotics in intra-abdominal infections. Antibiotics. 10.3390/antibiotics13080776 39200076 10.3390/antibiotics13080776 PMC11352101

[2] Coccolini F, Sartelli M, Sawyer R, Rasa K, Viaggi B, Abu-Zidan F, Soreide K, Hardcastle T, Gupta D, Bendinelli C, Ceresoli M, Shelat VG, ten Broek R, Baiocchi GL, Moore EE, Sall I, Podda M, Bonavina L, Kryvoruchko IA, Stahel P, Inaba K, Montravers P, Sakakushev B, Sganga G, Ballestracci P, Malbrain MLNG, Vincent J, Pikoulis M, Beka SG, Doklestic K, Chiarugi M, Falcone M, Bignami E, Reva V, Demetrashvili Z, Di Saverio S, Tolonen M, Navsaria P, Bala M, Balogh Z, Litvin A, Hecker A, Wani I, Fette A, De Simone B, Ivatury R, Picetti E, Khokha V, Tan E, Ball C, Tascini C, Cui Y, Coimbra R, Kelly M, Martino C, Agnoletti V, Boermeester MA, De’Angelis N, Chirica M, Biffl WL, Ansaloni L, Kluger Y, Catena F, Kirkpatrick AW (2023) Source control in emergency general surgery WSES, GAIS, SIS-E, SIS-A guidelines. World J Emerg Surg. 10.1186/s13017-023-00509-4 37480129 10.1186/s13017-023-00509-4 PMC10362628

[3] Peltan ID, McLean SR, Murnin E, Butler AM, Wilson EL, Samore MH, Hough CL, Dean NC, Bledsoe JR, Brown SM (2022) Prevalence, characteristics, and outcomes of emergency department discharge among patients with sepsis. JAMA Netw Open. 10.1001/jamanetworkopen.2021.47882 35142831 10.1001/jamanetworkopen.2021.47882 PMC8832179

[4] Humes DJ, Solaymani-Dodaran M, Fleming KM, Simpson J, Spiller RC, West J (2009) A population-based study of perforated diverticular disease incidence and associated mortality. Gastroenterology. 10.1053/j.gastro.2008.12.054 19185583 10.1053/j.gastro.2008.12.054

[5] Jamal Talabani A, Lydersen S, Endreseth BH, Edna T-H (2014) Major increase in admission- and incidence rates of acute colonic diverticulitis. Int J Colorectal Dis. 10.1007/s00384-014-1888-9 24802814 10.1007/s00384-014-1888-9 PMC4101254

[6] Cirocchi R, Popivanov G, Konaktchieva M, Chipeva S, Tellan G, Mingoli A, Zago M, Chiarugi M, Binda GA, Kafka R, Anania G, Donini A, Nascimbeni R, Edilbe M, Afshar S (2021) The role of damage control surgery in the treatment of perforated colonic diverticulitis: a systematic review and meta-analysis. Int J Colorectal Dis. 10.1007/s00384-020-03784-8 33089382 10.1007/s00384-020-03784-8 PMC8026449

[7] Waibel BH, Rotondo MF (2010) Damage control in trauma and abdominal sepsis. Crit Care Med. 10.1097/CCM.0b013e3181ec5cbe 20724875 10.1097/CCM.0b013e3181ec5cbe

[8] Sohn M, Iesalnieks I, Agha A, Steiner P, Hochrein A, Pratschke J, Ritschl P, Aigner F (2018) Perforated diverticulitis with generalized peritonitis: low stoma rate using a “damage control strategy.” World J Surg. 10.1007/s00268-018-4585-y 29541823 10.1007/s00268-018-4585-y

[9] Vermeulen J, Gosselink MP, Busschbach JJ, Lange JF (2010) Avoiding or reversing Hartmann’s procedure provides improved quality of life after perforated diverticulitis. J Gastrointest Surg. 10.1007/s11605-010-1155-5 20127201 10.1007/s11605-010-1155-5 PMC2836251

[10] Lambrichts DPV, Vennix S, Musters GD, Mulder IM, Swank HA, Hoofwijk AGM, Belgers EHJ, Stockmann HBAC, Eijsbouts QAJ, Gerhards MF, van Wagensveld BA, van Geloven AAW, Crolla RMPH, Nienhuijs SW, Govaert MJPM, di Saverio S, D’Hoore AJL, Consten ECJ, van Grevenstein WMU, Pierik REGJM, Kruyt PM, van der Hoeven JAB, Steup WH, Catena F, Konsten JLM, Vermeulen J, van Dieren S, Bemelman WA, Lange JF (2019) Hartmann’s procedure versus sigmoidectomy with primary anastomosis for perforated diverticulitis with purulent or faecal peritonitis (LADIES): a multicentre, parallel-group, randomised, open-label, superiority trial. Lancet Gastroenterol Hepatol. 10.1016/S2468-1253(19)30174-8 31178342 10.1016/S2468-1253(19)30174-8

[11] Faes S, Hübner M, Girardin T, Demartines N, Hahnloser D (2021) Rate of stoma formation following damage-control surgery for severe intra-abdominal sepsis: a single-centre consecutive case series. BJS Open. 10.1093/bjsopen/zrab106 34750614 10.1093/bjsopen/zrab106 PMC8576255

[12] Antoniou SA, Huo B, Ortenzi M, Anteby R, Tryliskyy Y, Carrano FM, Seitidis G, Mavridis D, Hoek VT, Serventi A, Bemelman WA, Binda GA, Duran R, Doulias T, Forbes N, Krancis NK, Grass F, Jensen J, Krogsgaard M, Massey LH, Morelli L, Oberkolfer CE, Popa DE, Schultz JK, Sultan S, Tuech JJ, Bonjer HJ (2024) EAES rapid guideline: surgical management of complicated diverticulitis - with ESCP participation. Surg Endosc. 10.1007/s00464-024-11445-y 39733172 10.1007/s00464-024-11445-y

[13] Jurt J, Hübner M, Clerc D, Curchod P, Abd El Aziz MA, Hahnloser D, Senn L, Demartines N, Grass F (2021) Challenges related to surgical site infection prevention-results after standardized bundle implementation. J Clin Med. 10.3390/jcm10194524 34640542 10.3390/jcm10194524 PMC8509330

[14] Giron H, Grass F, Hahnloser D (2025) Laparoscopic lavage: an option for surgical management of complicated diverticulitis. Surg Endosc. 10.1007/s00464-025-11617-4 40140084 10.1007/s00464-025-11617-4 PMC12041086

[15] Sohn MA, Agha A, Steiner P, Hochrein A, Komm M, Ruppert R, Ritschl P, Aigner F, Iesalnieks I (2018) Damage control surgery in perforated diverticulitis: ongoing peritonitis at second surgery predicts a worse outcome. Int J Colorectal Dis. 10.1007/s00384-018-3025-7 29536238 10.1007/s00384-018-3025-7

[16] Jacobson RA, Wienholts K, Williamson AJ, Gaines S, Hyoju S, van Goor H, Zaborin A, Shogan BD, Zaborina O, Alverdy JC (2020) *Enterococcus faecalis* exploits the human fibrinolytic system to drive excess collagenolysis: implications in gut healing and identification of druggable targets. Am J Physiol Gastrointest Liver Physiol. 10.1152/ajpgi.00236.2019 31604031 10.1152/ajpgi.00236.2019 PMC6985841

[17] Williams S, Bjarnason I, Hayee B, Haji A (2024) Diverticular disease: update on pathophysiology, classification and management. Frontline Gastroenterol. 10.1136/flgastro-2022-102361 38487561 10.1136/flgastro-2022-102361 PMC10935533

[18] Perathoner A, Klaus A, Mühlmann G, Oberwalder M, Margreiter R, Kafka-Ritsch R (2010) Damage control with abdominal vacuum therapy (VAC) to manage perforated diverticulitis with advanced generalized peritonitis–a proof of concept. Int J Colorectal Dis. 10.1007/s00384-010-0887-8 20148255 10.1007/s00384-010-0887-8

[19] Komen N, Dijk JW, Lalmahomed Z, Klop K, Hop W, Kleinrensink GJ, Jeekel H, Shouten WR, Lange JF (2009) After-hours colorectal surgery: a risk factor for anastomotic leakage. Int J Colorectal Dis. 10.1007/s00384-009-0692-4 19301016 10.1007/s00384-009-0692-4 PMC2689358

[20] Oberkofler CE, Rickenbacher A, Raptis DA, Lehmann K, Villiger P, Buchli C, Grieder F, Gelpke H, Decurtins M, Tempia-Caliera AA, Demartines N, Hahnloser D, Clavien PA, Breitenstein S (2012) A multicenter randomized clinical trial of primary anastomosis or Hartmann’s procedure for perforated left colonic diverticulitis with purulent or fecal peritonitis. Ann Surg. 10.1097/SLA.0b013e31827324ba 23095627 10.1097/SLA.0b013e31827324ba

[21] Cirocchi R, Afshar S, Shaban F, Nascimbeni R, Vettoretto N, Di Saverio S, Randolph J, Zago M, Chiarugi M, Binda GA (2018) Perforated sigmoid diverticulitis: Hartmann’s procedure or resection with primary anastomosis–a systematic review and meta-analysis of randomised control trials. Tech Coloproctol. 10.1007/s10151-018-1819-9 29995173 10.1007/s10151-018-1819-9

[22] Di Saverio S, Vennix S, Birindelli A, Weber D, Lombardi R, Mandrioli M, Tarasconi A, Bemelman WA (2016) Pushing the envelope: laparoscopy and primary anastomosis are technically feasible in stable patients with Hinchey IV perforated acute diverticulitis and gross faeculent peritonitis. Surg Endosc. 10.1007/s00464-016-4869-y 27005295 10.1007/s00464-016-4869-y

[23] Tadlock MD, Karamanos E, Skiada D, Inaba K, Talving P, Senagore A, Demetriades D (2013) Emergency surgery for acute diverticulitis: which operation? A national surgical quality improvement program study. J Trauma Acute Care Surg. 10.1097/TA.0b013e3182924a82 23694862 10.1097/TA.0b013e3182924a82

